# Effects of Vitamin D Receptor Genotype on Lipid Profiles and Retinopathy Risk in Type 2 Diabetes Patients: A Pilot Study

**DOI:** 10.3390/jpm12091488

**Published:** 2022-09-11

**Authors:** Hussam Alhawari, Yazun Jarrar, Dina Abulebdah, Sara J. Abaalkhail, Marah Alkhalili, Sura Alkhalili, Hussein Alhawari, Munther Momani, Mohammed N. Obeidat, Rand K. Fram, Mohammad A. Salahat, Su-Jun Lee

**Affiliations:** 1 Department of Internal Medicine, School of Medicine, The University of Jordan, Amman 11942, Jordan; 2 Department of Pharmaceutical Science, College of Pharmacy, Al-Zaytoonah University of Jordan, Amman 11733, Jordan; 3 Department of General Surgery, The University of Jordan Hospital, Amman 11942, Jordan; 4 Department of Pharmacology and Pharmacogenomics Research Center, College of Medicine, Inje University, Busan 50834, Korea

**Keywords:** diabetes, HDL, Jordanians, retinopathy, *VDR* gene

## Abstract

Genetic polymorphisms affect lipid profiles and are associated with disease complications. Genetic variants in the vitamin D receptor (VDR) gene are associated with type 2 diabetes mellitus (T2DM). In this study, we investigated the effects of *VDR* genotypes on the lipid profile and disease complications of T2DM patients in a Jordanian population. Ninety T2DM patients were genotyped for four major functional *VDR* genetic variants, rs2228570 C > T (*FokI*), rs7975232 A > C (*ApaI*), rs731236 T > C (*TaqI*), and rs1544410 C > T (*BsmI*), using the polymerase chain reaction–restriction fragment length polymorphism method. Lipid profiles and diabetes complications were analyzed and correlated with VDR genotypes. We found that the *VDR* rs7975232 and rs1544410 alleles were significantly (*p* = 0.008–0.04) associated with high-density lipoprotein (HDL) levels and retinopathy among patients. Carriers of the rs7975232 *A/A* genotype exhibited higher levels (49.68 ± 15.86 mg/dL) of HDL than patients with the *A/C* (44.73 ± 13.38 mg/dL) and *C/C* (37.93 ± 9.22 mg/dL) genotypes. Moreover, carriers of the rs1544410 *T/T* genotype had higher levels of HDL (54.31 ± 16.45 mg/dL) than patients with the *C/T* (43.57 ± 13.24 mg/dL) and *C/C* (43.98 ± 13.17 mg/dL) genotypes. T2DM patients who carry the rs7975232 *C/C* genotype were at higher risk (odds ratio [OR] = 7.88) of developing retinopathy compared with carriers of the rs7975232 *C/A* and *A/A* genotypes. In addition, T2DM patients with the rs1544410 *C/C* genotype had a higher risk (OR = 4.21) of developing retinopathy than patients with the rs1544410 *C/T* and *T/T* genotypes. Therefore, we concluded that the *VDR* rs7975232 and *rs1544410* alleles were associated with HDL levels and retinopathy and can be considered as potential genetic biomarkers for the lipid profile and retinopathy complication among T2DM patients in a Jordanian population of Arabic origin. Further studies with larger sample sizes are needed to confirm our findings.

## 1. Introduction

Diabetes mellitus (DM) is a chronic metabolic disease caused by insufficient insulin production, cellular insulin resistance, or both [1]. The long-term effects of diabetes can damage small (microvascular) and large (macrovascular) blood vessels throughout the body [2], which in turn harms the eyes, kidneys, nerves, and the cardiovascular system [3]. The lipid profile is disturbed in DM patients, characterized by elevated levels of triglycerides (TGs) and low-density lipoprotein (LDL), as well as low levels of high-density lipoprotein (HDL) [2]. These lipid changes can increase the cardiovascular risk of DM patients [3].

There are many risk factors for DM, including environmental and genetic factors. For example, genetic variants in inflammatory interleukins and the peroxisome proliferator-activator receptor are associated with type 2 diabetes mellitus (T2DM) and its complications [4,5].

Vitamin D plays a significant role in homeostasis; it reduces inflammation [6] and hyperlipidemia [7]. A recent study showed that vitamin D could modulate *Sars-Cov2* entry genes in the lungs [8]. Vitamin D deficiency is a cause of many disorders, including DM and dyslipidemia [9]. Vitamin D exerts its effects by binding the vitamin D receptor, a nuclear receptor that regulates the expression of many genes [10]. The vitamin D receptor (VDR) is encoded by the *VDR* gene located on chromosome 12 and is comprised of eight protein-encoding exons and six untranslated exons [11]. Genetic variants in the *VDR* gene can affect the function of the VDR and the vitamin D response [12]. The major functional *VDR* genetic variants are *FokI* (rs2228570, +30920 C > T), *ApaI* (rs7975232, +64978 A > C), *TaqI* (rs731236, +65058 T > C), and *BsmI* (rs1544410, +63980 C > T). The *FoxI* and *TaqI* variants are exonic, while the *ApaI* and *BsmI* variants are intronic. These *VDR* variant alleles have been linked to the levels of, and response to, vitamin D, and are associated with inflammatory and immune diseases, such as asthma and DM [13,14].

In a Jordanian study, Khdair et al. investigated the frequency of *VDR FokI*, *ApaI*, *TaqI*, and *BsmI* genotypes among type 1 diabetes mellitus (T1DM) patients [15. They found no difference in the frequency of these major *VDR* genotypes between TIDM patients and healthy volunteers [15]. To the best of our knowledge, there is no study regarding the influence of *VDR* genotype on T2DM among Jordanians. The aim of this study was to determine the effects of the *VDR FokI*, *ApaI*, *TaqI*, and *BsmI* genotypes on the lipid profile and major diabetes complications of T2DM patients of Jordanian Arabic origin.

## 2. Materials and Methods

### 2.1. Chemical Compounds

DNA primers were designed and obtained from Integrated DNA Technologies (Coralville, IA, USA). A Wizard genomic DNA extraction kit, 100-base pair (bp) ladder, and PCR master mix were purchased from Promega (Madison, WI, USA). Agarose gel and gel electrophoresis buffer (10× Tris-EDTA-borate buffer) were purchased from BioBasic (Markham, ON, Canada). RedSafe dye was purchased from iNtRON Biotechnology (Seongnam, South Korea).

### 2.2. Study Participants

**Figure 1:**
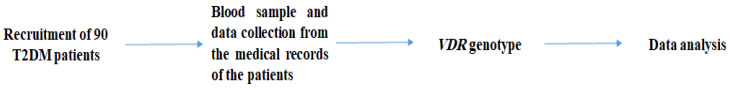
The flowchart of the research design.

The study design is summarized in Figure 1. Approximately 5 mL of blood was collected into EDTA tubes from 90 T2DM patients attending the University of Jordan Hospital from October 2021 to February 2022. The patients all had T2DM for ≥1 year and were diagnosed based on the 2021 diagnostic criteria of the American Diabetes Association (ADA) for T2DM (HbA1C% ≥ 6.5) [16]. The patients were under medical care for T2DM and medical records were available at the hospital. The patients were not on vitamin D therapy. The study protocol was explained in detail to all volunteers, and written informed consent was obtained.

The exclusion criteria were as follows: aged < 16 years, on vitamin D therapy, current pregnancy, and the presence of chronic comorbidities unrelated to diabetes complications, such as cancer, asthma, or rheumatoid arthritis. Ethical approval was provided by the Institutional Review Board of the University of Jordan Hospital (reference number: 129/2022).

### 2.3. Data Collection

Demographic data, blood lipid and glucose profiles, and total cholesterol, LDL, HDL, TG, and glycated hemoglobin (HbA1c%) data were obtained from the University of Jordan Hospital. Information regarding diabetes complications, such as cardiovascular disease (CVD), retinopathy, neuropathy, and nephropathy, were obtained from the patients’ medical records. The diagnosis of T2DM patients and identification of diabetes complications were performed by endocrinologists at the University of Jordan Hospital according to the guidelines of the ADA [16].

### 2.4. Genotyping of VDR Variants

Genomic DNA was extracted from each blood sample according to the manufacturer’s instructions using a Wizard DNA extraction kit. Then, the DNA samples were subjected to *VDR* gene amplification using a T100 thermal cycler (model number: 1861096; Bio-Rad, Hercules, CA, USA). Briefly, 100 ng of genomic DNA was added to a reaction mixture containing 10 pmol forward and 10 pmol reverse oligo DNA primers (Table 1), and 1 unit of *Taq* polymerase, in a standard buffer in a final volume of 50 µL. The PCR reaction conditions were as follows: 35 cycles of denaturation at 94 °C for 1 min, annealing at 57 °C for 1 min, and elongation at 72 °C for 1 min. To verify the amplification of *VDR* gene fragments, gel electrophoresis was carried out at 125 A for 30 min using 2.5% agarose gel. The bands were visualized following exposure to ultraviolet (UV) light with a wavelength of 302 nm using a benchtop UV transilluminator (BioDoc-Itt; Antylia Scientific, Vernon Hills, IL, USA). The expected sizes of the DNA fragments containing *VDR FokI*, *ApaI + TaqI*, and *BsmI* are 245, 745, and 823 bp (Supplementary Figure S1).

The *VDR* DNA fragments were subjected to restriction enzyme digestion using FoxI, ApaI, TaqI, and BsmI, to assess the *VDR FoxI*, *ApaI*, *TaqI*, and *BsmI* genotypes, respectively. Digestion was confirmed by gel electrophoresis using a 3.5% agarose gel. Digestion of the wild-type, heterozygous, and homozygous *VDR FoxI* genotypes produced a band of 245 bp; three bands of 245, 200, and 45 bp; and two bands of 200 and 45 bp, respectively (Supplementary Figure S2A). Digestion of the wild-type, heterozygous, and homozygous *VDR ApaI* genotypes produced a band of 745 bp; three bands of 745, 527, and 218 bp, and two bands of 527 and 218 bp, respectively (Supplementary Figure S2B). Digestion of the wild-type, heterozygous, and homozygous *VDR TaqI* genotype produced two bands of 497 and 248 bp; four bands of 497, 296, 248, and 201 bp; and three bands of 296, 248, and 201 bp, respectively (Supplementary Figure S2C). Digestion of wild-type, heterozygous, and homozygous *VDR BsmI* genotypes produced two bands of 638 and 185 bp; three bands of 823, 638, and 185 bp; and a single band of 823 bp, respectively (Supplementary Figure S2D).

### 2.5. In-Silico Analysis of the Promoter Sequence

Vitamin D response elements (VDREs) were predicted in the promoter sequences of the human *APOA1* gene, which encodes the major protein in HDL, using the online tool PROMO (version 8.3) and TRANSFAC database [17]. The DNA sequence of the promoter region of the human *APOA1* gene was obtained from the Eukaryotic Promoter Database (https://epd.epfl.ch//index.php, accessed on 29 July 2022) [18]. The promoter DNA sequence used for in-silico prediction corresponds to 1000 nucleotides before the transcriptional start site in the 5′-flanking region of the human *APOA1* gene.

### 2.6. Statistical Analysis

Statistical analyses were performed using SPSS software (version X7; IBM Corp., Armonk, NY, USA). The normality of the biochemical data was tested using the Kolmogorov–Smirnov test. Deviation of the frequency of the *VDR* genotype from Hardy–Weinberg equilibrium, and the frequency data for diabetes complications, were analyzed using the Chi-square test. One-way analysis of variance (ANOVA) followed by Tukey’s HSD post-hoc test was used to analyze the lipid profiles. The Kruskal–Wallis test was used to determine differences in the levels of HbA1C% and creatinine according to *VDR* genotype. *p*-values ˂ 0.05 were considered statistically significant.

## 3. Results

### 3.1. Patient Data

Blood cell data, biochemical parameters, lipid profiles, and information regarding diabetes complications were collected from 90 patients (males, n = 40; females, n = 50; Table 2). The mean ± standard deviation (SD) age of the participants was 63.43 ± 13.62 years. The average levels of LDL, HDL, and TGs were 105.17 ± 41.16, 45.69 ± 14.18, and 165.28 ± 86.29 mg/dL, respectively (Table 2). The average HbA1c and creatinine levels were 7.57 ± 1.71% and 0.95 ± 0.49 mg/dL, respectively.

More than half of the patients (55.6%) were suffering from CVD. In addition, 11.1% had retinopathy complications while 13.3% had chronic kidney diseases. Lastly, 17.8% of the patients exhibited diabetic neuropathy.

### 3.2. VDR Genotypes of T2DM Patients

Table 3 shows the frequency of the *VDR FoxI*, *ApaI*, *TaqI*, and *BsmI* genotypes among this sample of T2DM patients. The frequencies of the wild-type, hetero-, and homozygous *FoxI* genotypes were 45.1%, 43.30%, and 11.18%, respectively. The allele frequency of the *FoxI* C > T allele was 32%. The frequencies of the wild-type, hetero-, and homozygous *ApaI* genotypes were 27.8%, 57.8%, and 11.1%, respectively. The allele frequency of the *ApaI A > C* allele was 41%. The frequencies of the wild-type, hetero-, and homozygous *TaqI* genotypes were 41.1%, 42.2%, and 13.3%, respectively. The allele frequency of the *TaqI* T > C allele was 36%. Lastly, the frequencies of the wild-type, hetero-, and homozygous *BsmI* genotypes were 30%, 52.2%, and 17.8%, respectively. The allele frequency of the *BsmI C > T* allele was 44%. All frequencies of *VDR* variant alleles were in Hardy–Weinberg equilibrium, with *p*-values > 0.05 (Chi-square test).

### 3.3. VDR Haplotype and Linkage Disequilibrium among T2DM Patients

**Figure 2:**
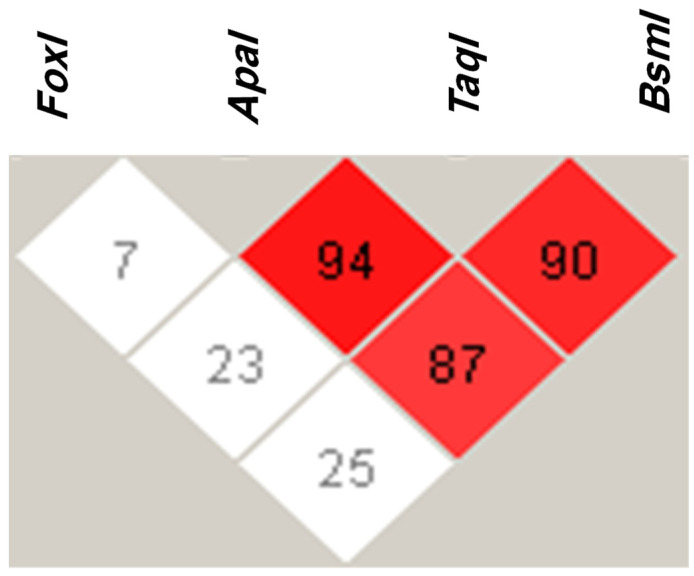
Linkage disequilibrium (LD) of the *FokI* (rs2228570 C > T), *ApaI* (rs7975232 A > C), *TaqI* (rs731236 T > C), and *BsmI* (rs1544410 C > T) *VDR* alleles in type 2 diabetes mellitus patients. LD was determined using Haploview software. Red squares represent a strong LD, and white squares a weak LD. The blue square indicates no LD.

The most common *VDR* haplotype was *FokI T*, *ApaI C*, *TaqI T*, and *BsmI C* with a frequency of 27% (Table 4). *FokI T*, *ApaI A*, *TaqI C*, and *BsmI T* was the second most common *VDR* haplotype, with a frequency of 25%. The least common *VDR* haplotype was *FokI T*, *ApaI A*, *TaqI C*, and *BsmI C*, with a frequency of 1% (Table 4). We found that *ApaI* was in strong linkage disequilibrium (LD) with the *TaqI* variant, with a D’ of 94 (Figure 2). In addition, *ApaI* was in LD with the *BsmI* variant, with a D’ of 87. LD was also evident between the *TaqI* and *BsmI* variants, with a D’ of 90. Lastly, the *FokI* variant was not in LD with the other *VDR* variants studied (Figure 2).

### 3.4. Associations of VDR Genotypes with Lipid, Glycemic, and Creatinine Levels

Table 5 shows the associations of the *VDR FokI*, *ApaI*, *TaqI*, and *BsmI* genotypes with lipid, glycemic, and creatinine levels in Jordanian T2DM patients. Neither the *FokI* nor *TaqI* genotype was associated with lipid, glycemic, or creatinine levels among T2DM patients. However, the *ApaI* and *BsmI* genotypes were significantly (*p* = 0.02–0.03) associated with HDL levels. Carriers of the wild-type *ApaI A/A* genotype had significantly higher levels (49.68 ± 15.86 mg/dL) of HDL than patients with heterozygous *ApaI A/C* (44.73 ± 13.38 mg/dL) and homozygous *ApaI C/C* (37.93 ± 9.22 mg/dL) genotypes. Carriers of the *BsmI T/T* genotype had significantly higher levels of HDL (54.31 ± 16.45 mg/dL) than those with the *BsmI C/C* (43.57 ± 13.24) or *BsmI T/C* (43.98 ± 13.17 mg/dL) genotype.

### 3.5. Associations of VDR Genotypes with Diabetes Complications

The associations of *VDR FoxI*, *ApaI*, *Taq1*, *and BsmI* genotypes with T2DM are illustrated in Table 6. *VDR FoxI* and *TaqI* genotypes were not associated with diabetes complications. On the other hand, the *ApaI* and *BsmI* genotypes were both significantly associated with retinopathy. Patients with the *ApaI C/C* genotype had a significantly (*p* = 0.008) higher risk of developing retinopathy compared with patients with the *ApaI A/C* and *A/A* genotypes. In addition, T2DM patients with the *BsmI T/T* genotype had a significantly (*p* = 0.04) higher risk of developing retinopathy compared with those with the *BsmI T/C* or *C/C* genotype.

### 3.6. In-Silico Prediction of VDREs in Promoter Sequences

Table 7 shows the results of predictions of VDREs in the promoter sequence of the human *APOA1* gene. Two different VDRE sequences, ACCC and GGGT, were identified at multiple positions in the *APOA1* gene promoter.

## 4. Discussion

Variants in the *VDR* gene influence the binding of vitamin D to its receptor, thereby affecting responses to vitamin D, and ultimately causing physiological disorders [19]. In this study, we investigated the influence of major *VDR* genotypes on lipid profiles and diabetes complications in a sample of T2DM patients of Jordanian Arabic origin. This study is the first done regarding the influence of *VDR* genotype on T2DM of the Jordanian Arabic population. The *VDR* variants *ApaI A > C* and *BsmI C > T* were significantly associated with HDL levels and retinopathy. This suggests the possibility that the *VDR* variants *ApaI C > A* and *BsmI C > T* could be used as genetic biomarkers for retinopathy and HDL levels among T2DM patients of Jordanian Arabic origin.

The average levels of HbAc1 and lipid profiles of the diabetes patients in this study were close to the targeted values, after diabetes medications, depending on the guideline of the American Diabetes Association [16]. This could be because our T2DM patients were on diabetic medications, such as metformin and glimepiride, as well as antihyperlipidemic drugs such as statins [20,21]. These medications may have affected the associations of *VDR* genotypes with biochemical profiles and diabetes complications.

The major T2DM complication among our patients was cardiovascular complications, consistent with previous studies of T2DM patients [22,23]. The prevalence of retinopathy (11.1%) in this study was lower than that reported by Al-Amer et al. (34%) in another study of Jordanians with T2DM [24]. However, the frequencies of diabetes complications observed in this study were within the ranges reported globally [25].

The present study is not the first study conducted in Jordan to investigate the influence of *VDR* genotypes on diabetes. Khdair et al. analyzed the frequency of *VDR* genotypes and haplotypes among T1DM patients and compared these frequencies with those of healthy controls [15]. Similar findings were observed in terms of *VDR* genotype, haplotype, and LD among our T2DM patients: the frequency of *VDR FoxI*, *ApaI*, and *Taq1* genotypes observed in T2DM patients was similar to that reported previously among T1DM and healthy volunteers; the haplotypes *VDR FoxI T/ApaI C/Taq1 T/BsmI C* and *FoxI T/ApaI A/Taq1 C/BsmI T* were the major *VDR* haplotypes in T2DM patients, as well as T1DM patients and healthy controls; and the *VDR ApaI*, *TaqI*, and *BsmI* variants were in strong LD, while *FoxI* was in weak LD, with other *VDR* variants in T1DM and T2DM patients. However, the frequency of the homozygous *VDR BsmI* genotype among the T2DM patients in this study (0.18) was significantly (Chi-square test, *p* value < 0.05) higher than that reported by Khdair et al. in T1DM patients (0.06) [15]. Further studies of the frequency of *VDR* genotypes and haplotypes among both T1DM and T2DM patients in Jordan are needed, with larger patient cohorts.

Several studies have reported associations of *VDR* variants with lipid profiles [26,27,28]. The *VDR ApaI* and *BsmI* genotypes were only associated with HDL levels in our T2DM patients. The *VDR* genotype has previously been shown to significantly influence HDL levels in T2DM patients [29,30]. On the other hand, some studies found no association between *VDR* genotypes and HDL levels [31,32]. We analyzed (in silico) the promoter region of the *APOA1* gene, which encodes the major protein in HDL, and found several binding sites for the VDR in the promoter region; this indicates that the human *APOA1* gene is influenced by vitamin D, and that genetic variants in *VDR* may influence the molecular response to vitamin D on the transcription of the *APOA1* gene, as well as HDL levels. Further studies are needed to determine the effects of vitamin D and *VDR* variants on RNA and protein levels of APOA1 and HDL.

In the present study, the *VDR ApaI* and *BsmI* genotypes were associated with retinopathy in T2DM patients. Patients with the homozygous *ApaI C/C* genotype had a 7.88-fold increased risk of developing retinopathy compared with patients with the *ApaI A/A* and *BsmI T/T* genotypes, while patients with the homozygous *BsmI C/C* genotype had a 4.21-fold increased risk. Assis et al. also reported an association of *VDR* variants with diabetic retinopathy in T2DM [31]. The *BsmI* allele was also associated with diabetic retinopathy in Korean DM patients [33]. The mechanism underlying the role of *VDR ApaI A > C* and *BsmI C > T* variants in diabetic retinopathy remains unclear. However, in the present study, the *ApaI* and *BsmI* variants were associated with both HDL levels and retinopathy. Therefore, the increased risk of retinopathy among patients with *VDR ApaI* and *BsmI* variant alleles may be related to the significant variation in HDL levels. In the recent NO BLIND study, Sasso et al. suggested that HDL levels are a risk factor for retinopathy in DM patients [34].

The results of this study do not support previous reports in which *VDR* variants were identified as risk factors for the development of renal-, neuro-, and cardiovascular complications [35,36,37]. The discordance between our results and previous studies may be due to differences in study design, or to ethnic differences between Jordanians and other studied populations, such as Asians and Europeans.

There were some limitations to this study. First, the sample size was relatively small and this study is considered a pilot study. Therefore, further clinical genetic studies with larger samples are needed to confirm our findings. Second, certain rare *VDR* variant genotypes were not included in this study. Sequencing the entire *VDR* gene and identifying its structure in T2DM patients will be important. Lastly, the blood levels of vitamin D were not analyzed, which could have helped elucidate the correlations of *VDR* genotype with vitamin D levels, diabetes complications, and lipid profiles.

## 5. Conclusions

The *VDR ApaI* and *BsmI* genotypes are associated with HDL levels and retinopathy among T2DM patients of Jordanian Arabic origin. Further clinical genetic studies with larger sample sizes are needed to confirm the findings of this study.

## Figures and Tables

**Table 1:** Primer sequences and annealing temperatures used for PCR amplification of the *VDR* gene.

Primer	Primer Sequence (5′-3′)	Size (bp)
FokI F	AGTTGGCCCTGGCACTGACTCTGCTCT	245
FokI R	ATGGAAACACCTTGCTTCTTCTCC CTC	
ApaI-TaqI F	CAGAGCATGGACAGGGAGCAA	745
ApaI-TaqI R	GCAACTCCTCATGGCTGAGGTCTC	
BsmI F	AACCAGCGGGAAGAG GTCAAGGG	823
BsmI R	CAACCAAGACTACAAGTACCGCGTCAG	

F, forward primer; R, reverse primer; bp, base pair.

**Table 2:** Demographic and clinical data of the diabetic patients.

Demographic Data	Value
Age (mean ± SD)	63.43 ± 13.62 years
Sex (%)	40 (44.4) males, 50 (44.6) females
Biochemical parameters (mean ± SD)	
HbcA1	7.57 ± 1.71%
LDL	105.17 ± 41.16 mg/dL
HDL	45.69 ± 14.18 mg/dL
TG	165.28 ± 86.29 mg/dL
Creatinine	0.95 ± 0.49 mg/dL
Diabetic complications (%)	
Cardiovascular disease	55 (55.6)
Retinopathy	10 (11.1)
Nephropathy	12 (13.3)
Neuropathy	16 (17.8)

**Table 3:** Frequencies of *VDR FoxI*, *ApaI*, *Taq1*, and *BsmI* genotypes among T2DM patients.

	VDR Genetic Variants			
Genotype	FokI	ApaI	Taq1	BsmI
Wild-type	41 (45.61%)	25 (27.8%)	37 (41.1%)	27 (30%)
Heterozygous	39 (43.30%)	52 (57.8%)	38 (42.2%)	47 (52.2%)
Homozygous	10 (11.18%)	10 (11.1%)	12 (13.3%)	16 (17.8%)
Missing data ^a^	0	3	3	0
Allele frequency				
Observed	0.32	0.41	0.36	0.44

^a^ The missing data are unclear *VDR* genotyping results.

**Table 4:** Frequency of *VDR FoxI*, *Apa1*, *Taq1*, and *BsmI* haplotypes among diabetic patients.

VDR Variant				
FokI	ApaI	Taq1	BsmI	Frequency ^a^
T	C	T	C	0.27
T	A	C	T	0.25
C	A	C	T	0.13
T	C	C	T	0.10
C	C	C	T	0.08
C	C	T	C	0.08
T	C	C	C	0.04
C	A	C	C	0.02
C	C	C	C	0.02
T	A	C	C	0.01

^a^ The frequency of each haplotype was calculated using the Haploview program.

**Table 5:** Associations of *VDR FokI*, *ApaI*, *Taq1*, and *BsmI* variants with the biochemical parameters of T2DM patients.

VDR Variant	Biochemical Parameter				
FokI genotype	HbA1C (%)	LDL (mg/dL)	HDL (mg/dL)	TG (mg/dL)	Creatinine (mg/dL)
Wild-type	7.75 ± 2.04	106.51 ± 39.15	47.17 ± 14.14	184.07 ± 102.8	0.98 ± 0.57
Heterozygous	7.34 ± 1.41	100.85 ± 38.13	43.91 ± 13.98	149.17 ± 69.98	0.93 ± 0.41
Homozygous	7.76 ± 1.25	116.50 ± 59.79	46.6 ± 15.81	151.20 ± 55.29	0.87 ± 0.41
*p*-value	0.54	0.55	0.58	0.17	0.73
ApaI genotype	HbA1C (%)	LDL (mg/dL)	HDL (mg/dL)	TG (mg/dL)	Creatinine (mg/dL)
Wild-type	7.94 ± 2.16	113.2 ± 40.19	49.68 ± 15.86	177.48 ± 86.83	1.05 ± 0.69
Heterozygous	7.5 ± 1.55	104.31 ± 41.64	44.73 ± 13.38	161.32 ± 90.57	0.92 ± 0.40
Homozygous	7.11 ± 1.39	104.93 ± 36	37.93 ± 9.22	165.42 ± 54.67	0.92 ± 0.34
*p*-value	0.39	0.66	0.03 *	0.52	0.52
Taq1 genotype	HbA1C (%)	LDL (mg/dL)	HDL (mg/dL)	TG (mg/dL)	Creatinine (mg/dL)
Wild-type	7.26 ± 1.48	108.71 ± 42.86	44.47 ± 12.02	161.65 ± 73.17	0.88 ± 0.33
Heterozygous	7.65 ± 1.53	104.21 ± 37.12	44.53 ± 13.51	165.96 ± 101.94	1.02 ± 0.64
Homozygous	8.45 ± 2.62	109.58 ± 45.75	50.92 ± 20.44	178.41 ± 75.22	0.98 ± 0.35
*p*-value	0.11	0.87	0.34	0.85	0.47
BsmI genotype	HbA1C (%)	LDL (mg/dL)	HDL (mg/dL)	TG (mg/dL)	Creatinine (mg/dL)
Wild-type	7.28 ± 1.55	104.01 ± 39.34	43.57 ± 13.24	154.23 ± 72.39	0.77 ± 0.23
Heterozygous	7.59 ± 1.49	106.38 ± 41.13	43.98 ± 13.17	168.54 ± 97.95	1.00 ± 0.46
Homozygous	8.00 ± 2.44	103.56 ± 46.11	54.31 ± 16.45	174.43 ± 72.97	1.08 ± 0.77
*p*-value	0.41	0.96	0.02 *	0.71	0.08

* Indicates a significant difference (*p*-value < 0.0), according to one-way ANOVA of lipid parameters and Kruskal–Wallis test of HbA1C% and creatinine levels.

**Table 6:** Associations of *VDR FoxI*, *ApaI*, *Taq1*, *and BsmI* variants with diabetes complications.

*VDR* Variant	Diabetes Complication							
*FoxI* genotype	Cardiovascular disease		Neuropathy		Retinopathy		Nephropathy	
	yes	no	yes	no	yes	no	yes	no
Wild-type	25	16	9	32	5	36	5	36
Heterozygous	21	18	5	34	5	34	5	34
Homozygous	4	6	2	8	0	10	2	8
*p*-value	0.47		0.56		0.49		0.80	
*ApaI* genotype	Cardiovascular disease		Neuropathy		Retinopathy		Nephropathy	
	yes	no	yes	no	yes	no	yes	no
Wild-type	13	12	7	18	3	22	3	22
Heterozygous	32	20	9	43	3	49	6	46
Homozygous	4	6	0	10	4	6	3	7
*p*-value	0.40		0.15		0.008 *		0.29	
*Taq1* genotype	Cardiovascular disease		Neuropathy		Retinopathy		Nephropathy	
	yes	no	yes	no	yes	no	yes	no
Wild-type	19	18	3	34	6	31	5	32
Heterozygous	24	14	11	27	2	36	5	33
Homozygous	6	6	2	10	1	11	1	11
*p*-value	0.53		0.08		0.29		0.89	
*BsmI* genotype	Cardiovascular disease		Neuropathy		Retinopathy		Nephropathy	
	yes	no	yes	no	yes	no	yes	no
Wild-type	14	13	3	24	6	21	4	23
Heterozygous	29	18	9	38	2	45	6	41
Homozygous	7	9	4	12	2	14	2	14
*p*-value	0.41		0.48		0.04 *		0.96	

* Indicates a significant difference (*p*-value < 0.05), according to the Chi-square test.

**Table 7:** VDREs in the promoter sequence of the human *APOA1* gene.

Gene	VDRE Position in the Promoter #	VDRE Sequence
Human *APOA1*	−5 to −8	ACCC
	−341 to −344	GGGT
	−735 to −738	ACCC
	−741 to −744	ACCC
	−746 to −749	ACCC
	−795 to −798	ACCC
	−802 to −805	ACCC
	−956 to −959	ACCC

# The position of VDREs in the promoter sequence is relative to the transcriptional start site of the human *APOA1* gene.

## Data Availability

The data are available with the corresponding authors upon request.

## Supplementary Materials

The following supporting information can be downloaded at: https://www.mdpi.com/article/10.3390/jpm12091488/s1, Figure S1: The PCR products of *VDR* gene fragments containing *Fox1* (A), *ApaI* and *TaqI* (B), and *BsmI* (C); Figure S2: Genotyping of VDR major genetic alleles using PCR-FRLP.

## References

[1] Kharroubi A.T., Darwish H.M. (2015). Diabetes mellitus: The epidemic of the century. World J. Diabetes.

[2] Hirano T. (2018). Pathophysiology of Diabetic Dyslipidemia. J. Atheroscler. Thromb..

[3] Basa A.L., Garber A.J. (2001). Cardiovascular disease and diabetes: Modifying risk factors other than glucose control. Ochsner. J..

[4] Khodaeian M., Enayati S., Tabatabaei-Malazy O., Amoli M.M. (2015). Association between Genetic Variants and Diabetes Mellitus in Iranian Populations: A Systematic Review of Observational Studies. J. Diabetes Res..

[5] Kruzliak P., Haley A.P., Starcevic J.N., Gaspar L., Petrovic D. (2015). Polymorphisms of the peroxisome prolifera-tor-activated receptor-gamma (rs1801282) and its coactivator-1 (rs8192673) are associated with obesity indexes in subjects with type 2 diabetes mellitus. Cardiovasc. Diabetol..

[6] Aranow C. (2011). Vitamin D and the Immune System. J. Investig. Med..

[7] Surdu A., Pînzariu O., Ciobanu D.-M., Negru A.-G., Căinap S.-S., Lazea C., Iacob D., Săraci G., Tirinescu D., Borda I. (2021). Vitamin D and Its Role in the Lipid Metabolism and the Development of Atherosclerosis. Biomedicines.

[8] Alruwaili M.A., Jarrar Y. (2022). Effects of vitamin C and D on the mRNA expression of angiotensin converting enzyme 2 receptor, cathepsin L, and transmembrane serine protease in the mouse lungs. Libyan J. Med..

[9] Li Y., Tong C.H., Rowland C.M., Radcliff J., Bare L.A., McPhaul M.J., Devlin J.J. (2021). Association of changes in lipid levels with changes in vitamin D levels in a real-world setting. Sci. Rep..

[10] Pike J.W., Meyer M.B. (2010). The Vitamin D Receptor: New Paradigms for the Regulation of Gene Expression by 1,25-Dihydroxyvitamin D3. Endocrinol. Metab. Clin. N. Am..

[11] Marozik P., Rudenka A., Kobets K., Rudenka E. (2021). Vitamin D Status, Bone Mineral Density, and VDR Gene Poly-morphism in a Cohort of Belarusian Postmenopausal Women. Nutrients.

[12] Vishnupriya S., Bindu C.H., Annamaneni S., Reddy K.P. (2011). Association of vitamin D receptor gene start codon (Fok1) polymorphism with high myopia. Oman J. Ophthalmol..

[13] Kılıç S., Sılan F., Hız M.M., Işık S., Ögretmen Z., Özdemir O. (2016). Vitamin D Receptor Gene BSMI, FOKI, APAI, and TAQI Polymorphisms and the Risk of Atopic Dermatitis. J. Investig. Allergy Clin. Immunol..

[14] Khan A., Khan S., Aman A., Ali Y., Jamal M., Rahman B., Ahmad M., Aasim M., Jalil F., Shah A.A. (2019). Associa-tion of VDR Gene Variant (rs1544410) with Type 2 Diabetes in a Pakistani Cohort. Balkan J. Med. Genet..

[15] Khdair S.I., Jarrar Y.B., Jarrar W. (2021). Immunogenetic Prediction of VDR Gene SNPs: Lack of Association with Sus-ceptibility to Type 1 Diabetes in Jordanian Patients. Diabetes Metab. Syndr. Obes..

[16] American Diabetes A. (2020). 2. Classification and Diagnosis of Diabetes: Standards of Medical Care in Diabetes-2020. Diabetes Care.

[17] Farré D., Roset R., Huerta M., Adsuara J.E., Roselló L., Albà M.M., Messeguer X. (2003). Identification of patterns in biological sequences at the ALGGEN server: PROMO and MALGEN. Nucleic Acids Res..

[18] Périer R.C., Junier T., Bonnard C., Bucher P. (1999). The Eukaryotic Promoter Database (EPD): Recent developments. Nucleic Acids Res..

[19] Sirajudeen S., Shah I., Al Menhali A. (2019). A Narrative Role of Vitamin D and Its Receptor: With Current Evidence on the Gastric Tissues. Int. J. Mol. Sci..

[20] Alhawari H., Jarrar Y., Alkhatib M.A., Alhawari H., Momani M., Zayed A., Alkamhawi R., Zihlif M. (2020). The Association of 3-Hydroxy-3-Methylglutaryl-CoA Reductase, Apolipoprotein E, and Solute Carrier Organic Anion Genetic Variants with Atorvastatin Response among Jordanian Patients with Type 2 Diabetes. Life.

[21] Abed E., Jarrar Y., Alhawari H., Abdullah S., Zihlif M. (2021). How the cytochrome 7a1 (CYP7A1) and ATP-binding cassette G8 (ABCG8) genetic variants affect atorvastatin response among type 2 diabetic patients attending the University of Jordan Hospital. Int. J. Clin. Pharmacol. Ther..

[22] Nathan D.M. (1993). Long-Term Complications of Diabetes Mellitus. N. Engl. J. Med..

[23] Wu H., Norton V., Cui K., Zhu B., Bhattacharjee S., Lu Y.W., Wang B., Shan D., Wong S., Dong Y. (2022). Di-abetes and Its Cardiovascular Complications: Comprehensive Network and Systematic Analyses. Front Cardiovasc. Med..

[24] Al-Amer R.M., Khader Y., Malas S., Abu-Yaghi N., Al-Bdour M., Ajlouni K. (2008). Prevalence and risk factors of dia-betic retinopathy among Jordanian patients with type 2 diabetes. Digit J. Ophthalmol..

[25] Bos M., Agyemang C. (2013). Prevalence and complications of diabetes mellitus in Northern Africa, a systematic review. BMC Public Health.

[26] Jia J., Tang Y., Shen C., Zhang N., Ding H., Zhan Y. (2018). Vitamin D receptor polymorphism rs2228570 is significantly associated with risk of dyslipidemia and serum LDL levels in Chinese Han population. Lipids Health Dis..

[27] Al-Daghri N.M., Mohammed A.K., Al-Attas O.S., Ansari M.G.A., Wani K., Hussain S.D., Sabico S., Tripathi G., Alokail M.S. (2017). Vitamin D Receptor Gene Polymorphisms Modify Cardiometabolic Response to Vitamin D Supplementation in T2DM Patients. Sci. Rep..

[28] Karonova T., Grineva E., Belyaeva O., Bystrova A., Jude E.B., Andreeva A., Kostareva A., Pludowski P. (2018). Rela-tionship Between Vitamin D Status and Vitamin D Receptor Gene Polymorphisms with Markers of Metabolic Syndrome Among Adults. Front Endocrinol..

[29] Xia Z., Hu Y., Han Z., Gao Y., Bai J., He Y., Zhao H., Zhang H. (2017). Association of vitamin D receptor gene pol-ymorphisms with diabetic dyslipidemia in the elderly male population in North China. Clin. Interv. Aging.

[30] Al-Daghri N.M., Al-Attas O.S., Alkharfy K.M., Khan N., Mohammed A.K., Vinodson B., Ansari M.G.A., Alenad A., Alokail M.S. (2014). Association of VDR-gene variants with factors related to the metabolic syndrome, type 2 diabetes and vitamin D deficiency. Gene.

[31] Assis C.S., Diniz T.G., Alcantara J.O.S., Brito V., do Nascimento R.A.F., Nunes M., Silva A.S., de Queiroga Evangelista I.W., Viturino M.G.M., de Lima R. (2022). Metabolic impact of the VDR rs1544410 in diabetic retinopathy. PLoS ONE.

[32] Mackawy A.M., Badawi M.E. (2014). Association of vitamin D and vitamin D receptor gene polymorphisms with chronic inflammation, insulin resistance and metabolic syndrome components in type 2 diabetic Egyptian patients. Meta Gene.

[33] Hong Y.J., Kang E.S., Ji M.J., Choi H.J., Oh T., Koong S.-S., Jeon H.J. (2015). Association between *Bsm1* Polymorphism in Vitamin D Receptor Gene and Diabetic Retinopathy of Type 2 Diabetes in Korean Population. Endocrinol. Metab..

[34] Sasso F.C., Pafundi P.C., Gelso A., Bono V., Costagliola C., Marfella R., Sardu C., Rinaldi L., Galiero R., Acierno C. (2019). High HDL cholesterol: A risk factor for diabetic retinopathy? Findings from NO BLIND study. Diabetes Res. Clin. Pract..

[35] Nosratabadi R., Arababadi M.K., Salehabad V.A., Shamsizadeh A., Mahmoodi M., Sayadi A.R., Kennedy D. (2010). Polymorphisms within exon 9 but not intron 8 of the vitamin D receptor are associated with the nephropathic complication of type-2 diabetes. Int. J. Immunogenet..

[36] Yang L., Wu L., Fan Y., Ma J. (2017). Vitamin D receptor gene polymorphisms in association with diabetic nephropathy: A systematic review and meta-analysis. BMC Med. Genet..

[37] Soroush N., Radfar M., Hamidi A.K., Abdollahi M., Qorbani M., Razi F., Esfahani E.N., Amoli M.M. (2016). Vitamin D receptor gene FokI variant in diabetic foot ulcer and its relation with oxidative stress. Gene.

