# Supplementary material for: Effects of Vitamin D Receptor Genotype on Lipid Profiles and Retinopathy Risk in Type 2 Diabetes Patients: A Pilot Study
Source: J Pers Med. 2022 Sep 11;12(9):1488. doi: 10.3390/jpm12091488 (PMC9505147; doi:10.3390/jpm12091488)
Supplement: Supplementary file 1 [file jpm-12-01488-s001.zip › jpm-1890072-supplementary.pdf]

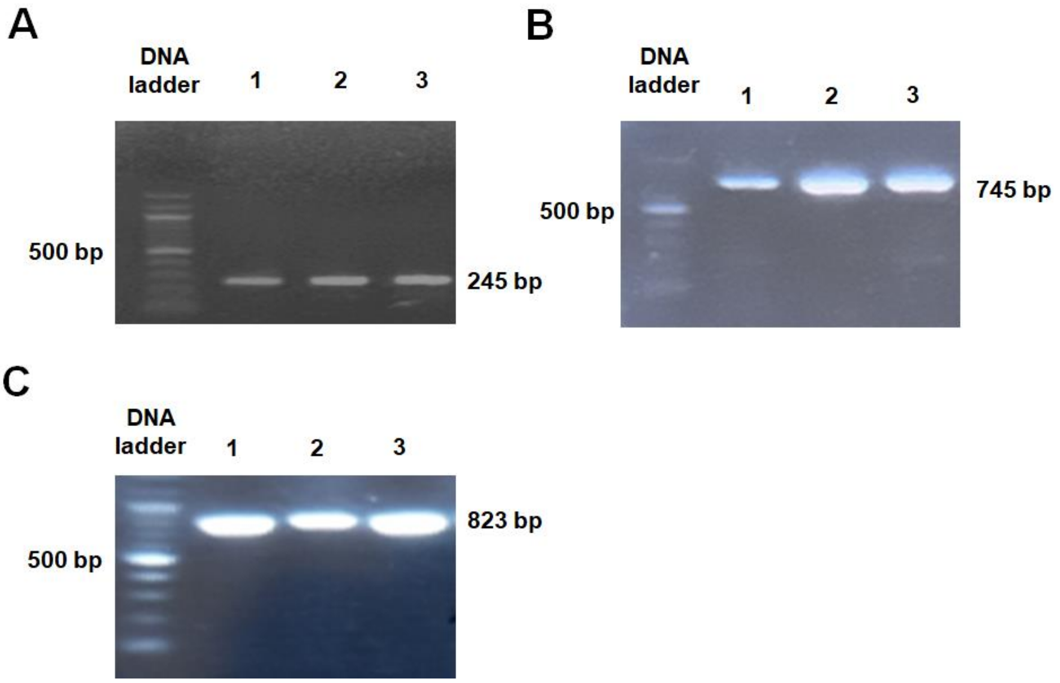

Supplementary Figure S1. The PCR products of *VDR* gene fragments containing *Fox1* (A), *ApaI* and *TaqI* (B), and *BsmI* (C). The numbers 1 to 3 represents random samples of patients used in this study.

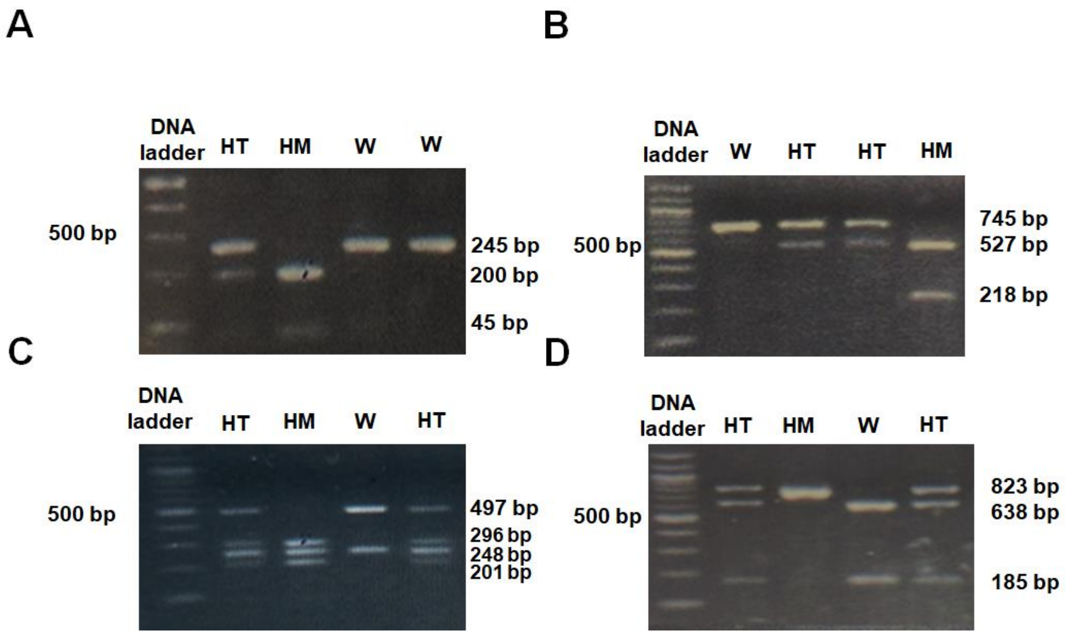

Supplementary Figure S2. Genotyping of *VDR* major genetic alleles using PCR-FRLP. (A) represents the genotype of *FoxI*, (B) represents the genotype of *ApaI*, (C) represents the genotype of *TaqI*, and (D) represents the genotype of *BsmI* genetic allele. "W" is the abbreviation of the wild genotype, "HT" is the abbreviation of heterozygous genotype, and "HM" is the abbreviation of homozygous genotype.
